# Nanoparticles in association with antimicrobial peptides (NanoAMPs) as a promising combination for agriculture development

**DOI:** 10.3389/fmolb.2022.890654

**Published:** 2022-08-23

**Authors:** Mariana Rocha Maximiano, Thuanny Borba Rios, Marcelo Lattarulo Campos, Guilherme Souza Prado, Simoni Campos Dias, Octávio Luiz Franco

**Affiliations:** ^1^ S-Inova Biotech, Pós-Graduação em Biotecnologia, Universidade Católica Dom Bosco, Campo Grande, Brazil; ^2^ Centro de Análises Proteômicas e Bioquímicas, Programa de Pós-Graduação em Ciências Genômicas e Biotecnologia, Universidade Católica de Brasília, Brasília, Brazil; ^3^ Integrative Plant Research Laboratory, Departamento de Botânica e Ecologia, Instituto de Biociências, Universidade Federal de MT, Cuiabá, Brazil; ^4^ Embrapa Arroz e Feijão, Laboratório de Biotecnologia, Goiânia, GO, Brazil; ^5^ Pós-graduação em Biologia Animal, Instituto de Biologia, Universidade de Brasília, Brasília, DF, Brazil

**Keywords:** antimicrobial peptides, nanostructure, crop production, biotic stress, food production, biotechnology, agribusiness

## Abstract

Antimicrobial peptides are small molecules, up to 10 kDa, present in all kingdoms of life, including in plants. Several studies report that these molecules have a broad spectrum of activity, including antibacterial, antifungal, antiviral, and insecticidal activity. Thus, they can be employed in agriculture as alternative tools for phytopathogen and pest control. However, the application of peptides in agriculture can present challenges, such as loss of activity due to degradation of these molecules, off-target effects, and others. In this context, nanotechnology can offer versatile structures, including metallic nanoparticles, liposomes, polymeric nanoparticles, nanofibers, and others, which might act both in protection and in release of AMPs. Several polymers and biomaterials can be employed for the development of nanostructures, such as inorganic metals, natural or synthetic lipids, synthetic and hybrid polymers, and others. This review addresses the versatility of NanoAMPs (Nanoparticles in association with antimicrobial peptides), and their potential applications in agribusiness, as an alternative for the control of phytopathogens in crops.

## 1 Introduction

Antimicrobial peptides (AMP) are small (up to 10 kDa) cationic molecules, with amphipathic structures composed of hydrophobic and positively charged domains (Bin Hafeez et al., 2021; Sarkar et al., 2021). These molecules have been found in all kingdoms of life. Contrary to what their name suggests, AMPs can present miscellaneous activities besides antimicrobial, including antiviral and insecticide (Huan et al., 2020; Erdem Buyukkiraz and Kesmen, 2021; Gera et al., 2021). AMPs can be employed to improve agriculture production, including diseases control. The biotic stress caused by pests and pathogens represents one of the main challenges to food security (Savary et al., 2019). Biotic stress can lead to up to 40% yield losses in our main crops, especially in food-deficit regions with fast-growing populations (Oerke, 2005; Savary et al., 2019). Additionally, this scenario can be aggravated by current climate changes, which increase microbial infection risks and foliar herbivory (Chaloner et al., 2021; Hamann et al., 2021).

The development of crop varieties that are more resistant to diseases and pest attacks represents a fundamental step toward achieving food security worldwide (Dhankher and Foyer, 2018). In this context, AMPs (either exogenously applied or transgene expressed) represent two approaches for improving plant resistance to phytopathogens (Li J. et al., 2021). Although AMPs present potent activity and easy metabolization without adversely affecting food quality, some restrictions limit their application in agriculture, including high production cost, safety concerns related to toxicity, low stability during transport, and easy hydrolysis by proteases (Wang et al., 2016; Huan et al., 2020).

In this context, nanobiotechnology arises as an interface between nanotechnology and biotechnology (Amin et al., 2011). In this interdisciplinary research field, tools on the nanometer scale, such as nanodevices, nanoparticles, and other nanostructured systems can be employed in the development of biotech products and applied to solving problems involving biological sciences and their concerns, e.g., biocatalysis, biomedicine, and agriculture (Barabadi, 2017; Thiruvengadam et al., 2018; Worrall et al., 2018). Nanomaterials used as these tools can be made of inorganic metals, liposomes, polymers, nanofibers, and others (Duhan et al., 2017). Although nanoparticles (nanospheres or nanocapsules) are the most popular nanostructured systems, other types are also very useful in biotechnology, such as dendrimers, nanogels, and liposomes (Jiang et al., 2007).

All of these nanostructures, developed by several materials, can be employed as drug delivery systems (DDS) (Vega-Vasquez et al., 2020) due to their properties of harboring and/or attaching molecules of interest that will act in specific cells, tissues, or organs in a controlled release mechanism (Allen and Cullis, 2004; Jiang et al., 2007). This can makes nanomaterials very suitable vehicles for the gradual release of a wide range of molecules, including secondary metabolites, nucleic acids, proteins, and peptides. This release can improve the delivery at the desired target site, by addressing cells in a spatiotemporal manner (Martínez-Ballesta et al., 2018).

Taking this into account, nanotechnology has been used in the last few years to associate nanoscale delivery systems with AMPs (NanoAMPs) to stabilize these molecules. When compared to isolated AMPs, which have lower bioavailability and are usually unstable in the environment, the NanoAMPs can bypass this disadvantages and increase the biological effect on the target (Biswaro et al., 2018). NanoAMPs can also promote a controlled release of entrapped AMPs, therefore keeping a longer time of action, improving half-life time, decreasing potential toxicity, and promoting the biological activity in constant doses (Tan et al., 2021). Moreover, this association may be useful for enhancing the effectiveness of either AMPs or nanostructured systems themselves, or even boosting their activities synergistically through combinatorial formulations (León-Buitimea et al., 2020).

Among the main advantages of using NanoAMPs over free AMPs, it is possible to point out the AMP side-effects decrease, as lower bioavailability and the environment instability in the, lower administration frequency, a lower dose needed, constant levels of AMPs released, bioavailability enhanced by defense against degradation, maximization of biological activity, in addition to applying to a wide range of molecules (Patra et al., 2018). In this context, NanoAMPs can be an interesting alternative to bypass plant biotic stress and improve agricultural production.

## 2 A tool to achieve food security: The potential of antimicrobial peptides in agriculture

AMPs present several beneficial characteristics, including activity against several phytopathogens (fungi, bacteria, virus) and insects (Mulinari et al., 2007; Pinto et al., 2012); the capacity to generate direct and durable plant resistance; and small gene nature that facilitates stacking the coding sequence of multiple AMPs on single expression vectors (Islam et al., 2021). Besides, AMPs natural or synthetic also present ease of manipulation and optimization by computational approaches (*in silico* design). These approaches can includes, search by homology modeling, molecular dynamics and protein docking. The advantages of computational *in silico* methods include their low cost, faster procedure speed, simple process (Porto et al., 2018; Costa et al., 2020; Hashemi et al., 2021; Delaunay and Ha-Duong, 2022). These approaches can be employed i.e. to generation of derivatives with improved features, and a low metabolic cost of production (da Cunha et al., 2017; Porto et al., 2018), which reduces potential detrimental impacts on plant growth and productivity associated with the activation of the plant defense responses (Keymanesh et al., 2009; Campos et al., 2018; Sarkar et al., 2021).

Moreover, AMPs can be described as an eco-friendly and healthier alternative for controlling pest and pathogens (Li P. et al., 2021). By this way, several studies developed transgenic plants expressing AMPs, including rice, wheat, potato, tomato, banana and soybean to improve the resistance against biotic and abiotic stress (Supplementary Table S1). In summary these studies highlight that AMPs expression presents high potential to increase resilience to pests, pathogens and abiotic stress. Additionally, plants expressing these peptides can present a decrease in demand of chemical pesticides, that can cause risks to the environment and consumers’ health (Keymanesh et al., 2009).

In this context, are important highlights the limitations to AMP gene expression in plants and the challenges faced in development and commercialization of transgenic plant lines (Turnbull et al., 2021; Bakare et al., 2022; Sharma et al., 2022). Although plants are able to express antimicrobial peptides, some pitfalls can be faced, as the production of AMP in all plant structures, difference among expression and difficult to produce active plant AMPs in large quantities due the differences in plant cultivation, and the endogenous AMPs degradation by plant proteases (Bakare et al., 2022).

Furthermore, AMPs also can be applied in order to control plant diseases through non-transgenic methods, such as exogenous applications (i.e. spraying with or immersion in peptide solutions) and food coating (Wang et al., 2018a). Exogenous application of peptides PAF56 (GHRKKWFW) and cecropin A-melittin hybrid peptide BP21 (Ac-FKLFKKILKVL-NH2) in citrus can control post-harvest green mold, one of the main postharvest diseases, and blue mold and sour rot, caused respectively by *Penicillium digitatum*, *Penicillium italicum*, and *Geotrichum candidum* (Wang et al., 2018a; Wang et al., 2018b). Additionally, peptide O3TR and its derived lipopeptide C12O3TR were also employed to protect freshly harvested orange fruit against *P. digitatum* (Li et al., 2019).

Despite several studies indicating that AMPs stand out as a barrier to ward of phytopathogen and pest attacks, only a few studies have demonstrated positive applications in field conditions. This can be explained by challenges associated with upscaling production, or with stability of the peptides. Regarding AMP-derived plant resistance to biotic stress, few studies have moved from the laboratory to the most applicable field conditions, thus hampering our ability to use these peptides directly to protect agroecosystems (Huan et al., 2020). This situation may be explained by challenges usually associated with the production or activity of AMPs, including a reduction in defensive activity due to degradation of these molecules when in contact with microbial proteases or enzymes present in the digestive system of herbivores or due the environmental conditions such as sunlight, temperature and others, off-target effects leading to cytotoxicity to the consumer (in case of the transgenic plant) and high production costs for exogenous applications (Keymanesh et al., 2009; Biswaro et al., 2018; Huan et al., 2020). In this context, nanotechnology is now arising as a revolutionary and versatile alternative by which to optimize the biological and chemical properties of AMPs, and this may finally bring the benefits of these peptides to consumers.

## 3 Advantages of nanotechnology for antimicrobial peptide activity

Nanotechnology can be a promising alternative for the storage and administration of antimicrobial peptides, once nanostructures can protect AMPs from proteolysis and unwanted interactions and can promote a controlled, long-lasting, and targeted release of the peptide (Sandreschi et al., 2016). Additionally, these nanostructures have the potential to protect the AMP against environmental conditions such as sunlight, and variation in temperature, and others (Badea et al., 2015; López-Vargas et al., 2018; Felippim et al., 2020). Thus, NanoAMPs have been developed in recent years based on the association of nanoscale delivery systems with AMPs (Table 1).

**TABLE 1:** NanoAMPs based on association of nanoscale delivery systems and AMPs.

Antimicrobial peptides	Nanoparticle	Potential application (health/Agriculture)	Approach description	Application/Effects	References
polymyxin B	Silver nanoparticles	Health	*In vitro* assay to evaluation of synergism between polymyxin B and Silver nanoparticles	Antibiotic synergy against Gram-negative bacteria	Ruden et al. (2009)
P13	Silver nanoparticles	Health/Agriculture	*In vitro* assay to evaluation antibacterial activity, against both Gram-negative and Gram-positive bacteria, cytotoxicity against mouse fibroblast, and evaluation of physical chemical characteristics	Decrease in AgNP cytotoxicity, improvement in antimicrobial activity and in stability in aqueous solution	Gao et al. (2020)
HHC-8					
MM-10	Poly (ε-caprolactone) nanoparticles (PCL-NPs)	Health	*In vitro* assay to evaluation of the ability to protect encapsulated materials from proteolysis, AMP release by photothermal triggered, and effects in activity against Gram-negative and Gram-positive bacteria	AMP degradation protection and sustained release; and Improvement in antibiotic activity against *bacteria*	Moorcroft et al. (2020)
gramicidin A melittin Alamethicin	Lipidic inverse bicontinuous cubic phase nanoparticles (Cubosomes)	Health/Agriculture	*In vitro* assay to validation of systems for the delivery of AMPs	Validation of encapsulation systems for the delivery of AMPs	Meikle et al. (2017)
LL37	Silica nanoparticles	Health	*In vitro* assay to evaluation of roles of membrane interactions for the successful use of mesoporous silica nanoparticles as delivery systems for antimicrobial peptides (AMPs)	Delivery system and AMP degradation protection	Braun et al. (2016)
nisin	Microemulsions	Health/Agriculture	*In vitro assay* to evaluation of microemulsion based in different essential oil to encapsulate nisin enhancing the system’s overall antimicrobial activity	Activity against bacteria*,* in lettuce leafs	Chatzidaki et al. (2018)
P_5_VP_5_	Nanoparticle self-assemble	Agriculture	*In planta assay*	Reduction in the development of citrus canker lesions, inhibition of biofilm formation, damage to cell membranes, and effects on cell membrane permeability	Shuai et al. (2019)

These studies focus on human or animal health to stabilize these molecules compared to isolated AMPs, which have lower bioavailability and are usually unstable in the environment when used alone, thus reducing their biological effect on the target (Biswaro et al., 2018). Besides, NanoAMPs can promote a controlled release of entrapped AMPs, therefore maintaining a longer time of action, improving half-life time, decreasing potential toxicity and promoting biological activity in constant doses (Tan et al., 2021). Moreover, this association may be useful for enhancing the effectiveness of either AMPs or nanostructured systems themselves, or even boosting both their activities synergistically through combinatorial formulations (León-Buitimea et al., 2020).

In general, nanomaterials can be functionalized with AMPs, promoting the generation of NanoAMPs to bypass some challenges faced in AMP applications in agriculture including an increase in AMP stability, target activity, release of entrapped AMPs, biological activity and decreasing the potential toxicity of AMPs’ effects on the environment. Thus, NanoAMPs present great potential in agribusiness, considering their advantages and wide range of applications (Patra et al., 2018).

## 4 Nanoparticles in association with antimicrobial peptides: Promising applications of associating antimicrobial peptides with nanostructured systems

The development of nanometric structures complexed with bioactive molecules has shown a high impact in several areas, including agriculture (Duhan et al., 2017). This approach enables the controlled, efficient, and safe release of fertilizers, pesticides and herbicides in several plant crops (Santana et al., 2020; Zhang et al., 2020). Besides, nanotechnology employment in agriculture, has shown a role in increasing the abiotic stresses tolerance plants, including drought, heat, salinity and ion toxicity, oxidative stress and others. Studies that employed cerium oxide (CeO_2_) nanoparticles, called nanoceria showed the potent antioxidant properties that can decrease the drought-induced oxidative stress, by catalytic scavenging reactive oxygen species (ROS), in model plants well as in plants of interest economic (Wu et al., 2017; Djanaguiraman et al., 2018). Besides, the combination of nanoparticles with peptides can also be used for generating nanosensors capable of early stress detection (Zhang et al., 2011; Giraldo et al., 2019).

The wide-ranging potential of nanobiotechnology applications in agriculture can be related to the wide nanomaterials range employed in the nanoparticles development (Li J. et al., 2021). The nanomaterials differ in size, shape, composition, and physicochemical properties, and may vary in surface area and the reactivity of the molecule. These characteristics should promote an improvement in the solubility and half-life of the molecule, including AMPs, and a decrease in toxicity due to their ability to target the specific site of action (Reis et al., 2006; Bawa, 2009; Zhang et al., 2013). Additionally, different materials have been used for NanoAMP preparation, such as inorganic metals (Min et al., 2009; Goswami et al., 2010), liposomes (Luo et al., 2015), polymers (Rafiee et al., 2014; Kleine-Brueggeney et al., 2015) and nanofibers (Lahiani et al., 2015).

Metallic nanoparticles can be used as antimicrobial agents or nanocarriers for active substances. Among metallic nanoparticles, silver is known for its antimicrobial activity and is considered the most promising nanomaterial, mainly due to its bactericidal properties and adaptability to different substrates (Cho et al., 2005; Sharma et al., 2009). Moreover, silver has gained popularity due “green synthesis” production. These approaches involves metallic nanoparticles synthesis using bioactive agents including plants, bacteria and fungi to the bio reduction of metal ions in their elemental form, that presents size range 1–100 nm (Rafique et al., 2017; Rodrigues et al., 2021; Patil et al., 2022). The green synthesis depends on the employment of water solvent for nanoparticles yield. Bioreduction and biosorption are essential routs for that synthesis. Bioreduction can be described as the process in which metal ions are chemically reduced into their stable forms; and the biosorption process involves the binding of metal ions (generated by bioreduction) on the surface of bioactive agent (Gobalakrishnan et al., 2021; Kaur and Sidhu, 2021). The use of natural precursors for the biosynthesis of nanoparticles has some advantages when compared to conventional methods of synthesis, such as biocompatibility and low production costs, since these synthesis routes do not use toxic solvents or chemical precursors (El-Sherbiny and Salih, 2018; Gour and Jain, 2019). Other metal nanoparticles include copper, titanium dioxide, and gold, which are mostly used for the incorporation of fertilizers, with little research into disease management (Sadeghi et al., 2017).

NanoAMPs, developed using silver nanoparticles and AMPs, in general aim to deliver NanoAMPs to intracellular target sites and show lower cytotoxicity; additionally, enhanced AMP activity was observed in some studies (Ruden et al., 2009; Gakiya-Teruya et al., 2020; Gao et al., 2020; Zharkova et al., 2021). Concerning functionalization, the association of five different amphiphilic α-helical AMPs (PGLa, MSI-103, MAP, BP100, and TP10) with gold nanoparticles by attachment of the peptides to the gold core, exclusively via the N-terminal Cys, aimed to increase the stability of peptides against enzymes such as trypsin. This resulted in an improvement in the AMPs’ lifetime, antimicrobial activity against Gram-negative and positive bacteria, and stability towards trypsin action while AMPs maintained their conformational flexibility (Wadhwani et al., 2017). Additionally in biomedical studies, a PEG hydrogel was recently co-loaded with gold nanorods encapsulating the AMP named IK8. These nanoparticles ensuring IK8 proteolysis protection and release control. Consequently bactericidal activity was enhanced through photothermal activation based on laser irradiation (Moorcroft et al., 2020).

Liposomes are another nanostructure commonly applied in the protection of molecules. These nanostructures are spherical vesicles with an amphiphilic lipid bilayer membrane structure with mean diameters from nanometer to micrometer. Their properties, functionalities and stability depend on factors such as temperature, pH, ionic strength, concentration, and composition of phospholipids and the properties of the encapsulated molecule (Jesorka and Orwar, 2008). Liposomes are the most used drug delivery system and can be obtained from natural or synthetic lipids; an example is a phosphatidylcholine, which is one of the lipids most used in liposome formulation (Pinilla et al., 2021).

The application of liposomes is widely reported in several biomedical studies (Makowski et al., 2019; Wang et al., 2021). Additionally, in biomedical studies, a PEG hydrogel was recently co-loaded with gold nanorods encapsulating the AMP named IK8. These nanoparticles ensuring IK8 proteolysis protection and release control. Consequently bactericidal activity was enhanced through photothermal activation based on laser irradiation (Moorcroft et al., 2020). The usefulness of cubosomes (also called *lipidic inverse bicontinuous cubic phase nanoparticles*) as encapsulation systems for the delivery of AMPs has been validated (Meikle et al., 2017; Meikle et al., 2021). On the other hand, the role of liposomes in agriculture has been related to cell membrane model systems (Taylor et al., 2005; Isozumi et al., 2021), food preservation in the post-harvest process or industrial processing, and the protection of substances such as enzymes, vitamins, and antimicrobials, to improve food quality (Mozafari, 2005; da Silva Malheiros et al., 2010; Pinilla et al., 2021).

In this context, the plant application of NanoAMPs faces some challenges, including physical structures present in leaf, such as hair and cuticular wax which can be barriers to this approach. Nevertheless, nanoparticles obstruction depends on the physical characteristics such as particle size, epidermal structure, leaf area, and plant growth stage. Once the lipophilicity of leaf wax can promote the adsorption of hydrophobic or lipophilic nanoparticles, the nanoparticle material choice can be decisive to bypass such challenge (López-Vargas et al., 2018; Su et al., 2019; Hong et al., 2021).

Polymers are the main nanoparticles constituents used in drug delivery systems. Polymeric nanoparticles are formed by a polymeric matrix and can retain the molecule internally or adsorb to the polymeric structure (Vauthier and Bouchemal, 2009; Brandelli, 2012). They are more robust and stable particles than liposomes because they are held together by covalent bonds. Thus, several other polymeric nanoparticles have been used as vehicles for diverse AMPs with different applications, in several areas, including the health area. In this context, the influence of porosity and surface charge of mesoporous silica nanoparticles (MSN) on loading and release of AMP LL-37 was investigated and results showed that anionic mesoporous silica particles incorporated considerable amounts of LL-37 (cationic AMP). In addition, these particles protect LL-37 from degradation by proteases (Braun et al., 2016).

Nanofibers are one-dimensional nanomaterials produced with a wide range of natural, synthetic and hybrid polymers. As the name suggests, these are fiber-shaped nanomaterials with several unique properties such as nanoporosity, high surface area/volume and high mass transport properties (Meraz-Dávila et al., 2021). Factors such as temperature, viscosity, solution surface tension and electric field strength are important to nanostructures, but to nanofibers has larger importance, once define the quality and characteristics of these nanostructures (Deitzel et al., 2001).

Due to their property of high surface area to volume ratio, nanofibers have a great potential for carrier and release antimicrobials peptides. In addition, different modes of carrying molecules can be obtained; antimicrobials peptides can be loaded onto the surface of the nanofiber by adsorption, there may be adsorption of charged nanoparticles with the molecule on the surface of the fibers, or a layer-by-layer assembly on the cover allows some nanometers to deposit polyanions such as heparin (Yoo et al., 2009). Encapsulated synthetic AMP HHC-8 and MM-10 in poly (ε-caprolactone) nanoparticles (PCL-NPs), which triggered minimal degradation and sustained release of AMPs and improved their antimicrobial activity against mycobacteria, ensured the synergistic effect of NanoAMPs (Sharma et al., 2021). Thus, nanofibers present a strong potential for distributing antimicrobials in food systems.

## 5 Potential applications of nanoparticles in association with antimicrobial peptides in agriculture

The challenges faced by food production are distinct in pre-harvest and post-harvest phases. In pre-harvest phase, food production can be negatively affected by several phytopathogens. These organisms can include fungi, bacteria, viruses, parasites, and insects, which can cause the development of several diseases and promote several losses in food production. In the post-harvest phase, one of the most challenges faced is the losses caused by mold infections. In both scenarios, NanoAMPs can be a promising approach and offer different strategies/combinations to mitigate these problems. During pre-harvest, different NanoAMPs can be employed for disease control, and in post-harvest, the NanoAMPs can promote an increase in the shelf life of food, well as protection against mold infections (Brandelli, 2012; Duhan et al., 2017; Sadeghi et al., 2017; Biswaro et al., 2018; Ristaino et al., 2021; Rizzo et al., 2021; Singh et al., 2021).

**FIGURE 1:**
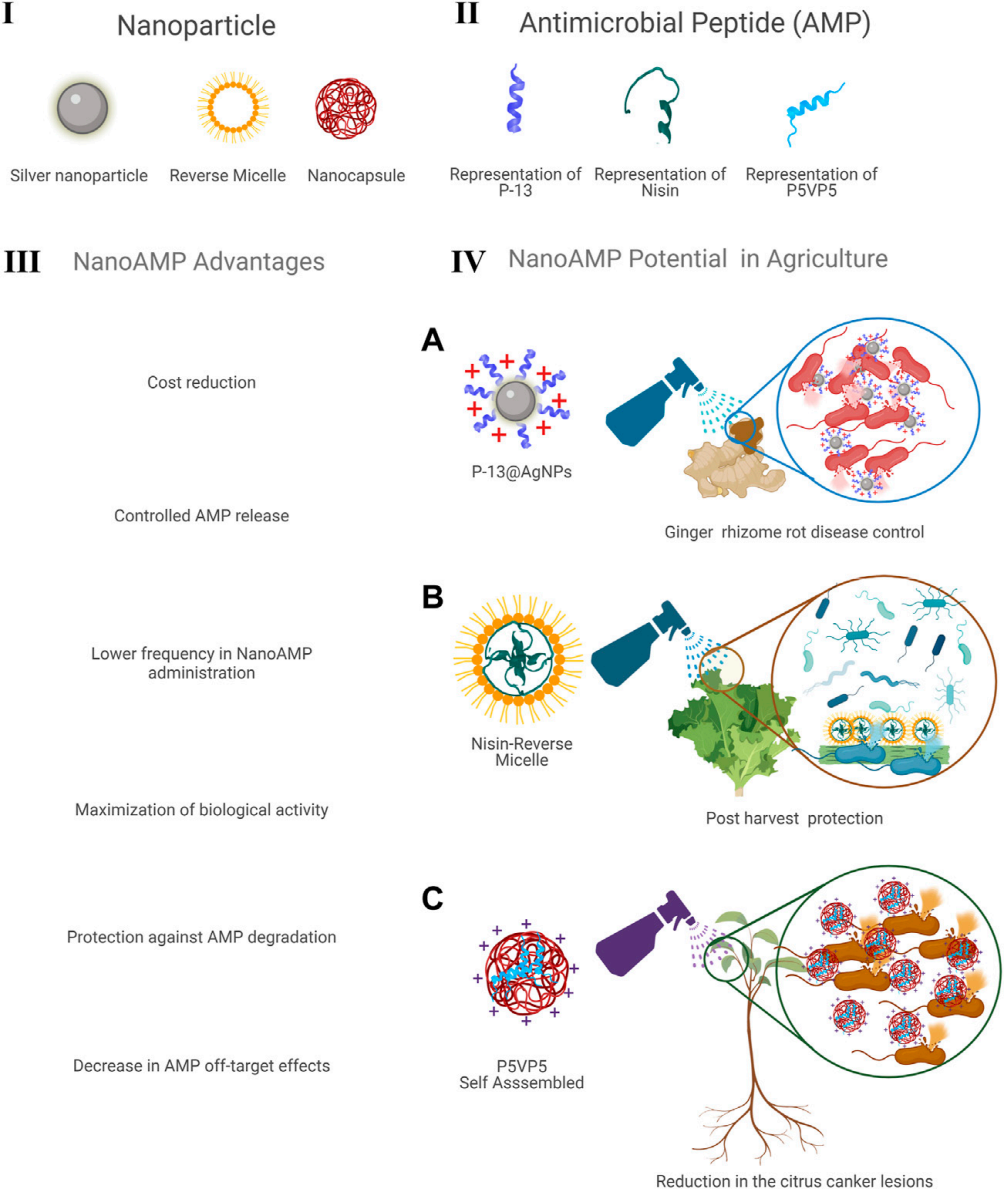
NanoAMPs description in which, nanoparticles (I) were associated with antimicrobial peptides (II) showing advantages (III) and potential applications in agriculture (IV). **(A)** Representation of P13@AgNPs, which presents potential to control ginger rhizome rot disease caused by *Bacillus pumilus*. **(B)** Representation of nisin peptide combined with reverse micelles that shows potential for post-harvest food protection. **(C)** Representation of self-assembled P5VP5 peptide that can be used to decrease citrus canker lesions caused by *Xanthomonas axonopodis* pv. *citri*. Figure developed with support of the Biorender (Biorender.com).

In this context, some NanoAMPs examples applied in agriculture can be highlighted (Figure 1), including a NanoAMP called P-13@AgNPs, achieved through the rational development of an antimicrobial peptide (P-13) and its association with silver nanoparticle (AgNP), which results in better activity against Gram-negative and Gram-positive bacteria (Gao et al., 2020), including activity toward *Bacillus pumilus,* which can cause ginger rhizome rot disease (Peng et al., 2013), demonstrating the potential application of this NanoAMP in plant disease control.

Additionally, NanoAMPs were developed using nisin (an important peptide in the food industry) in association with several lipid-based nanostructures, including liposomes, nanoemulsions, solid lipid nanoparticles (SLNs), and nanostructured lipid carriers (NLCs), have been employed in food conservation, and present potential for use in agriculture, specifically in post-harvest (Bahrami et al., 2019). These NanoAMPs can be employed in food preservation, since nisin presents activity against bacteria such as *Listeria monocytogenes* and *Lactobacillus plantarum* (Prombutara et al., 2012). However, a NanoAMP based on nisin associated with a nanoemulsion (reverse micelles through W/O microemulsions) presented antimicrobial activity, during an *in vitro* assay in lettuce fresh leaves (Chatzidaki et al., 2018), suggesting that these NanoAMPs can be employed in post-harvest.

Another interesting example of NanoAMPs employed in agriculture is the NanoAMP development called P_5_VP_5_. This peptide was engineered as a unique symmetrical cationic peptide $\left(\text{AC}-\overset{+}{R}L\left|\overset{+}{R}\overset{+}{K}\vee \overset{+}{K}\overset{+}{R}\right|L\overset{+}{R}-{\text{NH}}_{2}\right)$ , which was characterized by simple sequences and can readily form stable nanoparticles (self-assembled), and presents excellent thermal stability under various environmental conditions (Shuai et al., 2019). Additionally, the P_5_VP_5_ nanoparticle reduced the citrus canker lesions in the leaves of citrus plants. This disease is caused by *Xanthomonas axonopodis* pv. *citri*, and can be considered one of the most devastating diseases of citrus plants. This nanoparticle also presented activity against biofilm formation (Shuai et al., 2019).

## 6 Challenges and perspectives

The employment of NanoAMPs is still strongly focused on the biomedical field (Mohid and Bhunia, 2020), especially for therapeutic applications (Teixeira et al., 2020; Gera et al., 2021), and studies concerning applications in agribusiness are still scarce. Although several tools are already available for the development of this area, many applications remain unexplored, making more studies and research necessary to develop solid solutions for problems faced in agriculture as well as in livestock. Some researchers (Perez-de-Luque and Rubiales, 2009) have already proposed the use of nanotechnology strategies for parasitic plant control, suggesting the nanoencapsulation of herbicides to be used against parasitic weeds, and noticing the potential of nanoparticles as magic bullets for the delivery of herbicides, chemicals, nucleic acids, enzymes and even AMPs targeting specific plant tissues for the treatment of viruses and microbial parasites. Additionally, some studies evaluate the environmental impacts of nanomaterials and conclude that most of nanoparticles are unlikely to have adverse effects on human health or on environment (McClements and Xiao, 2017; Lead et al., 2018; Sohal et al., 2018). Furthermore, the development and deployment of nanoAMPs can be employed in protected agriculture, i.e in glasshouses (Sarika et al., 2012).

Thus, NanoAMPs certainly have a great potential in agribusiness, considering the wide variety of applications described here and the various benefits, mainly in the improvement of productivity and safety against microbial contaminants. However, NanoAMPs remain underdeveloped for agribusiness applications which development currently underway and no commercial NanoAMPs products available in the sector.

Finally, we must consider that substantial work has already been done toward using free AMPs in agriculture as discussed above, and the use of either AMPs for antimicrobial activity or food preservation (Keymanesh et al., 2009) also provides a complete review of many. This paves the way for intensive research dedicated to improving the supply of nutrients, pesticides, herbicides and food preservatives through nanotechnology approaches, using what has already been tested through free AMPs.
