# Supplementary material for: Nanoparticles in association with antimicrobial peptides (NanoAMPs) as a promising combination for agriculture development
Source: Front Mol Biosci. 2022 Aug 23;9:890654. doi: 10.3389/fmolb.2022.890654 (PMC9447862; doi:10.3389/fmolb.2022.890654)
Supplement: Supplementary file 1 [file Table1.docx]

**Supplementary table 1.** Transgenic plants expressing antimicrobial peptides.

| Antimicrobial peptides | Plant | Approach | Effects | References |
| --- | --- | --- | --- | --- |
| BP100 | Rice | Transgenic plants (constitutive expression) | Improvement in the resistance against bacterial pathogen *Dickeya chrysanthemi*, and against the fungus Fusarium verticillioides (biotic stress), additionally also improved the tolerance to oxidative stress (abiotic stress). | (Nadal et al., 2012) |
| Thanatin | Rice | Transgenic plants | Enhances resistance to rice blast, caused by fungus *Magnaporthe oryzae.* | (Imamura et al., 2010) |
| MsrA2 and 10R | Wheat | Transgenic plants | Decrease in fungi diseases susceptibility, such as head blight and powdery mildew disease. | (Badea et al., 2013) |
| SN1 | Wheat | Transgenic plants | Enhances resistance toward bacteria diseases, such as sharp eyespot, common root rot, spot blotch, and black point diseases. | (Wang et al., 2012) |
| Snakin-1 | Potato | Transgenic plants (overexpression) | Enhances resistance toward causal agents of stem canker, black scurf and, soft rot diseases. | (Almasia et al., 2008; Bártová et al., 2019) |
| *Capsicuum annum* (chili) defensin | Tomatoes | Transgenic plants | Improvement in the resistance toward fungal pathogens that can cause diseases as fusarium wilt and late blight respectively. | (Zainal et al., 2009) |
| Sm-AMP-D1 | Banana | Transgenic plants | Enhances resistance against fungal *Fusarium oxysporum*. | (Ghag et al., 2014) |
| CaAMP1 | Soybean | Transgenic plants | Increases in tolerance toward Phytophthora root and stem rot, caused by *Phytophthora spp*. | (Niu et al., 2020) |
